# Hydrodynamic Contrast Recanalization for Crossing Challenging Culprit Occlusions in Acute Myocardial Infarction

**DOI:** 10.1016/j.jaccas.2026.109200

**Published:** 2026-07-07

**Authors:** Amad Chohan, Angelo Nascimbene, Mauro Carlino, Anjula Chib, Hassan Ashraf, Syed Altamesh, Salman Arain

**Affiliations:** a Department of Cardiology, McGovern Medical School, University of Texas Health Science Center at Houston (UTHealth), Houston, Texas, USA; b Unità di Emodinamica, Department of Cardiology, IRCCS San Raffaele Scientific Institute, School of Medicine, Vita-Salute San Raffaele University, Milan, Italy

**Keywords:** chronic total occlusion, HDR, percutaneous coronary intervention, STEMI

## Abstract

Hydrodynamic contrast recanalization (HDR)—a technique adapted from chronic total occlusion percutaneous coronary intervention in which small-volume microcatheter contrast injection creates a low-resistance pathway for wire crossing—can facilitate crossing of challenging culprit occlusions in acute myocardial infarction. We present 3 cases in which conventional wire escalation failed but HDR enabled successful first-attempt crossing, each representing a distinct occlusion acuity: acute thrombotic, subacute organized, and chronic total occlusion. Early use of HDR when crossing proves difficult may reduce crossing time, minimize dissection risk, and reduce thrombus embolization.

## Graphical Abstract

**Figure:**
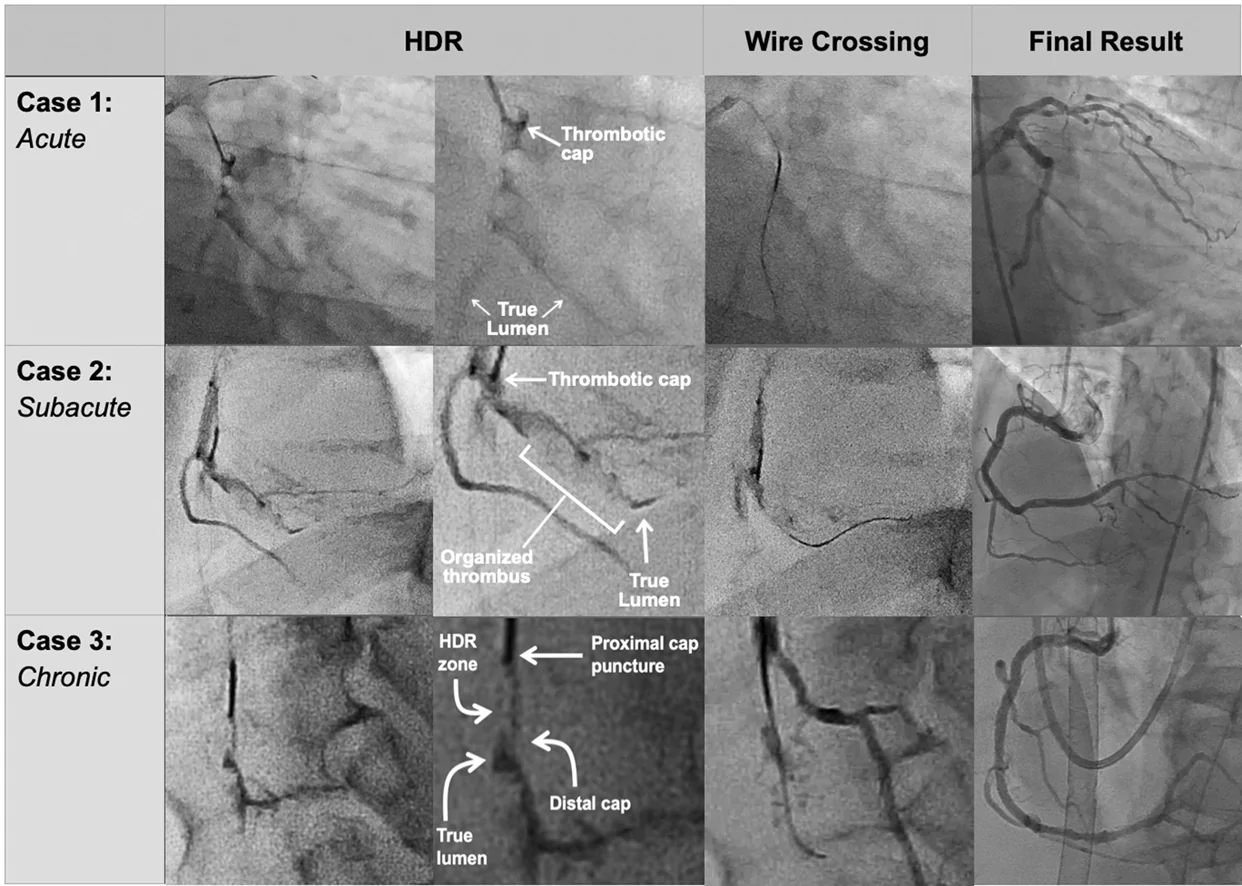

**Visual Summary:**
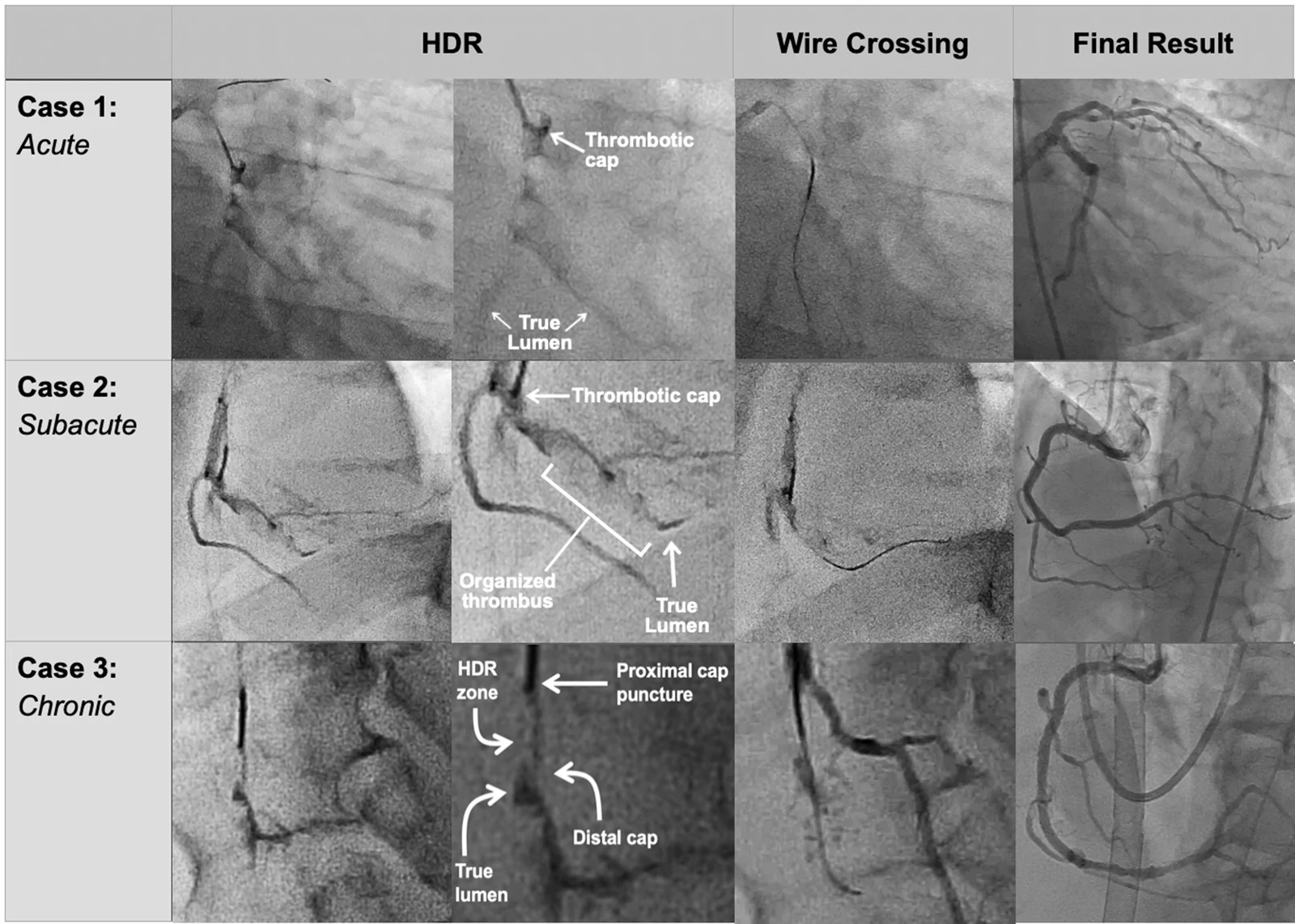
Central Illustration: Summary of Each Case Columns from left to right: HDR contrast injection, first-attempt wire crossing after HDR, and final angiographic result after stenting. (Top row, left to right) Case 1: HDR injection reveals a dense thrombotic cap with contrast staining traveling around the more organized thrombus into the distal circumflex and obtuse marginal. The Fielder XT wire crossed the lesion through the contrast-modified channel on the first attempt after HDR. Final angiography demonstrates TIMI flow grade 3 with no residual stenosis after drug-eluting stent implantation. (Middle row, left to right) Case 2: HDR injection demonstrates contrast following the path of least resistance through the extensively organized thrombus with identification of the true lumen of the distal RCA. The Mongo wire crossed this path into the distal vessel on the first attempt. Final angiography demonstrates TIMI flow grade 3 throughout the entire RCA after aspiration thrombectomy and drug-eluting stent implantation. (Bottom row, left to right) Case 3: HDR injection into the near-total occlusion of the mid-RCA with crossing of contrast into the distal RCA consistent with a type 2A staining pattern and identifying the distal cap of the short segment CTO. The Fielder XT wire crossed through the contrast-modified channel on the first attempt. Final angiography demonstrates TIMI flow grade 3 throughout the RCA after drug-eluting stent implantation. CTO = chronic total occlusion; HDR = hydrodynamic contrast recanalization; RCA = right coronary artery.

Hydrodynamic contrast recanalization (HDR) is a technique originally described in the context of chronic total occlusion (CTO) percutaneous coronary intervention (PCI), in which a small-volume controlled contrast injection through a microcatheter positioned in the proximal cap of an occlusion creates a low-resistance pathway for crossing the occlusion (Figure 1).^1^, ^2^, ^3^, ^4^, ^5^ Although the majority of culprit occlusions in ST-segment elevation myocardial infarctions (STEMIs) can be easily crossed with standard workhorse wires, some—particularly those with organized thrombus, large thrombus burden, or complex underlying plaque—resist conventional wiring strategies.^6^^,^^7^ In these cases, continued wire escalation risks vessel injury and has the potential to delay reperfusion. We present 3 cases demonstrating the application of HDR to primary PCI for acute myocardial infarctions (MIs), each with a culprit occlusion of different acuity: acute, subacute, and chronic.

**Figure 1:**
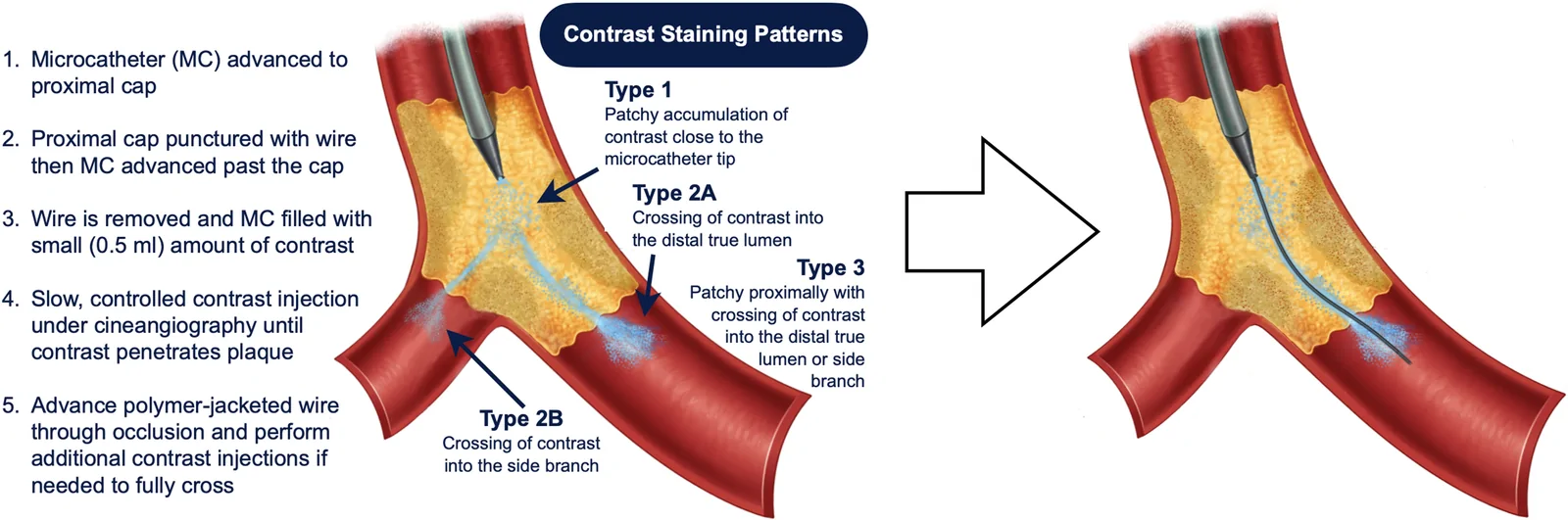
Hydrodynamic Contrast Recanalization Technique and Contrast Staining Patterns (Left) Hydrodynamic contrast recanalization is performed by advancing a microcatheter to the proximal cap of the occlusion, puncturing the cap with a polymer-jacketed wire, then advancing the microcatheter past the cap. After wire removal, the microcatheter is filled with a small volume of contrast (0.5 mL) and a slow, controlled injection is performed under cineangiography until contrast penetrates the plaque. The resulting contrast staining pattern characterizes the occlusion and facilitates subsequent wire advancement. Four staining patterns have been described: type 1, patchy contrast accumulation near the microcatheter tip, indicating a dense proximal occlusion without a clear distal channel; type 2A, contrast crossing into the distal true lumen, representing the most favorable pattern for wire crossing; type 2B, contrast crossing into a side branch, indicating the path of least resistance is toward a branch rather than the distal vessel; and type 3, patchy proximal staining with reconstitution of contrast into a channel to the distal true lumen or side branch, representing a mixed pattern. (Right) After injection, a polymer-jacketed wire is advanced through the contrast-prepared channel, crossing the occlusion into the distal true lumen. MC = microcatheter.

## Case Presentation

### Case 1: Acute occlusion—partially organized thrombus after plaque rupture

**Figure 2:**
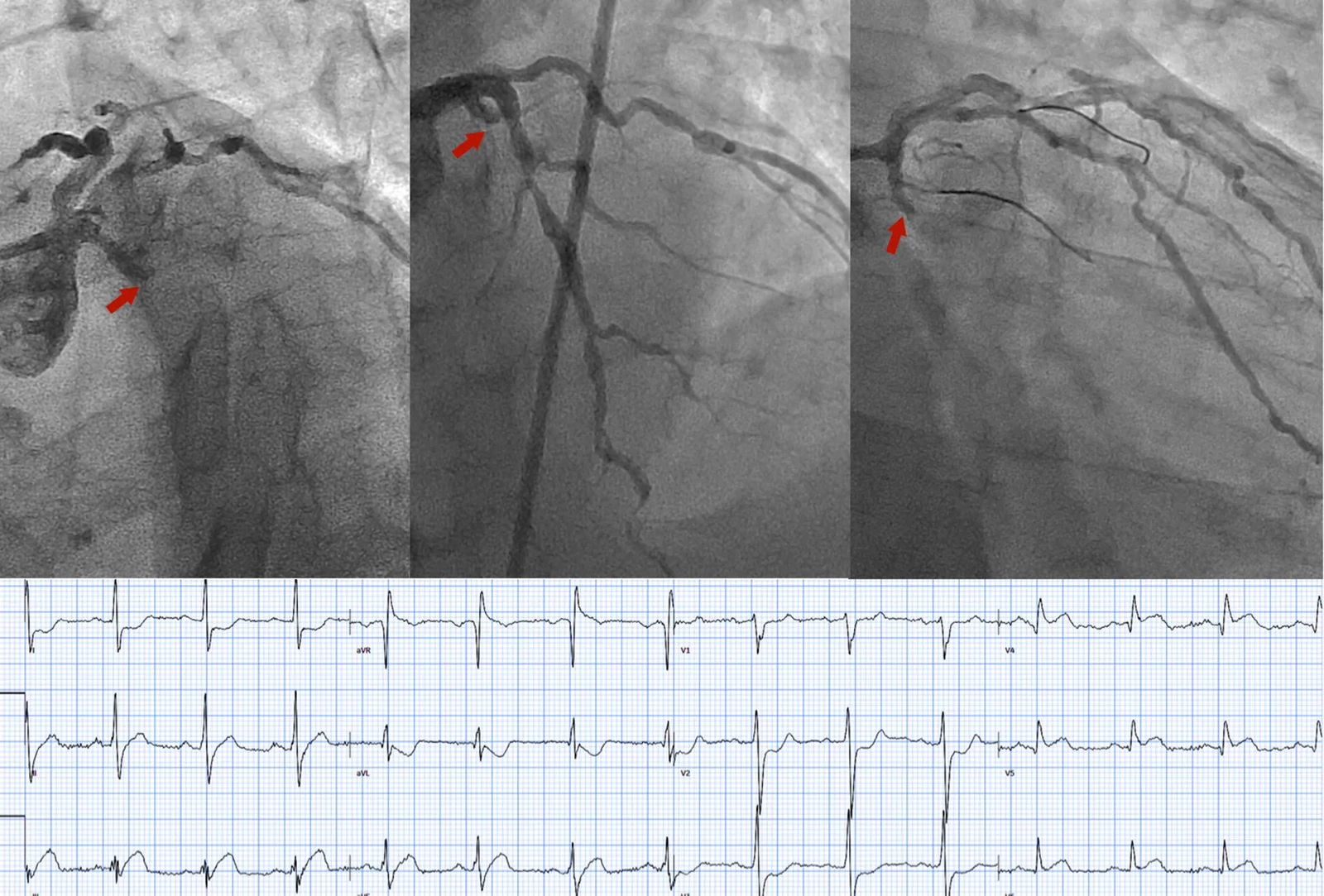
Case 1 Presenting Electrocardiogram and Diagnostic Coronary Angiography (Top) Coronary angiography demonstrating 100% occlusion of the proximal left circumflex artery (red arrows) in a large left-dominant system. (Bottom) Electrocardiogram obtained at the outside emergency department demonstrating inferior ST-segment elevations with posterior extension, consistent with a posterior STEMI due to left circumflex occlusion. STEMI = ST-segment elevation myocardial infarction.

A 53-year-old man with hypertension and hyperlipidemia developed acute-onset dyspnea and diaphoresis followed shortly by right arm weakness and numbness. He presented to an outside emergency department, where an electrocardiogram (ECG) demonstrated inferior ST-segment elevations with posterior extension (Figure 2). Given the concern for concurrent stroke, computed tomography angiography of the head was obtained, which demonstrated occlusion of the distal left internal carotid artery. Thrombolytics were deferred given the neurological deficits were nondisabling. The patient continued to have chest pressure and became hypotensive, requiring norepinephrine infusion up to 15 μg/min. Initial high-sensitivity troponin I was 351 ng/L, ultimately rising to >125,000 ng/L on subsequent measurement. He was transferred to a PCI-capable facility for emergent coronary angiography.

Coronary angiography performed approximately 5 hours after symptom onset demonstrated 100% culprit occlusion of the left circumflex artery (LCx) in a large left-dominant system. The left anterior descending artery (LAD) had severe chronic disease with focal 70% to 80% stenoses, and the right coronary artery (RCA) demonstrated an 80% midvessel stenosis (Figure 2). Attempts to cross the LCx occlusion with Runthrough (Terumo), Sion (Asahi), and Sion Black (Asahi) wires were unsuccessful, even with use of a Turnpike LP microcatheter (Teleflex) for support.

HDR was then performed as described in Figure 1 using a Fielder XT wire (Asahi) and a Turnpike LP microcatheter.^1^ The Fielder XT wire crossed the lesion on the first attempt. The microcatheter was advanced through the occlusion and the Fielder XT was exchanged for a Runthrough wire. Balloon angioplasty was performed followed by intravascular ultrasound (IVUS), which demonstrated a proximal LCx thrombotic plaque rupture with minimal calcification and a distal reference vessel diameter of approximately 4.25 mm. The lesion was prepared with cutting balloon angioplasty (3.5 × 10 mm Wolverine, Boston Scientific) followed by deployment of a 4.0 × 20 mm drug-eluting stent (Synergy, Boston Scientific) in the proximal LCx. Repeat IVUS confirmed adequate stent expansion with a minimum stent area of 13 mm^2^. Final angiography demonstrated restoration of TIMI flow grade 3 with no residual stenosis. Brain magnetic resonance imaging subsequently demonstrated left-sided watershed infarcts attributed to MI-related hypoperfusion in the setting of chronic internal carotid artery occlusion.

### Case 2: Subacute occlusion—extensively organized thrombus in a late-presenting MI

**Figure 3:**
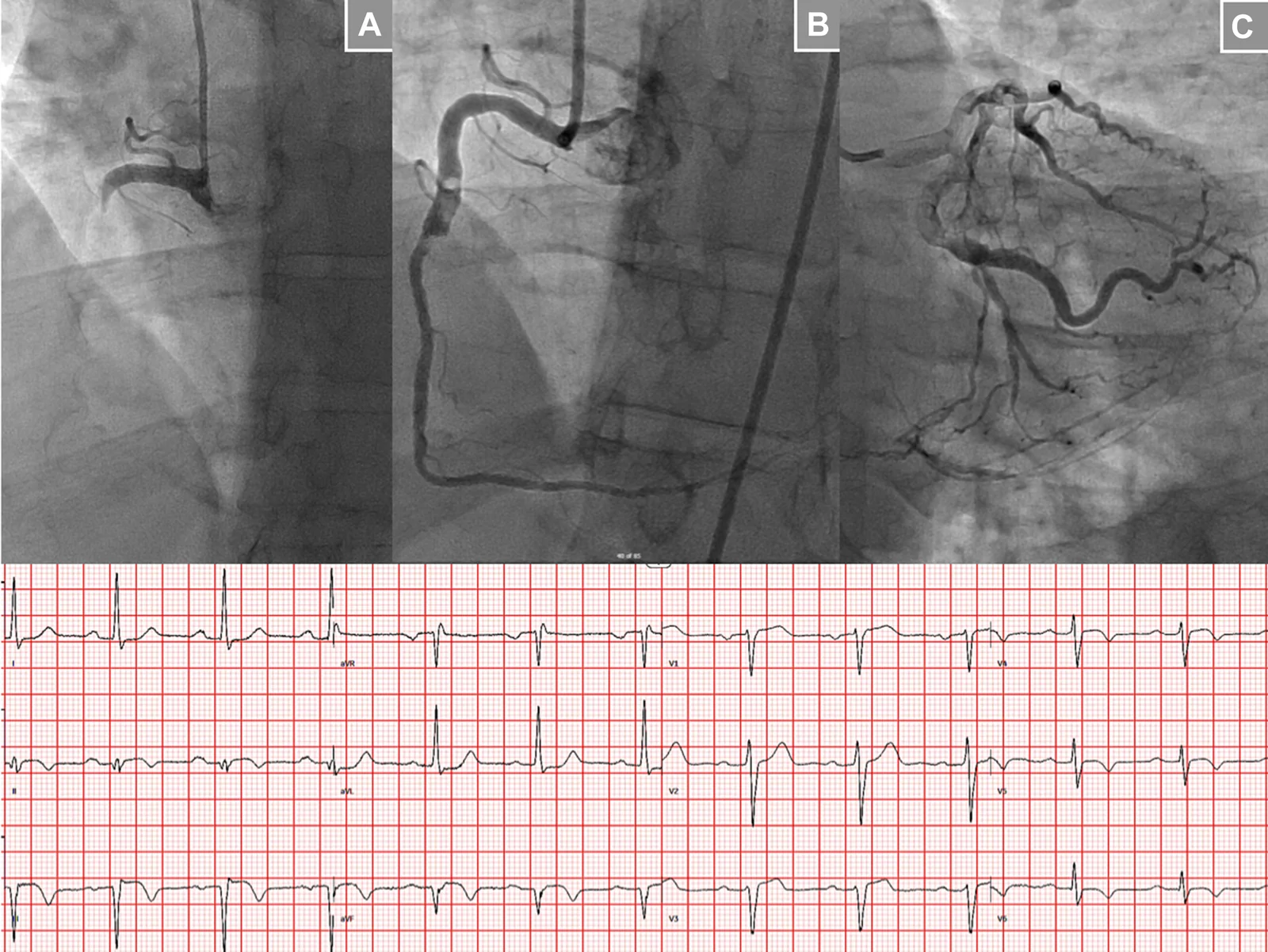
Case 2 Presenting Electrocardiogram and Initial Coronary Intervention (A) Baseline coronary angiography demonstrating 100% occlusion of the proximal right coronary artery with large thrombus burden and no distal vessel opacification. (B) Angiography following multiple passes of aspiration thrombectomy of the proximal RCA and RV marginal branch with an Export catheter demonstrating persistent thrombus burden. (C) Left coronary angiography demonstrating retrograde collateral filling of the distal right coronary artery from the left anterior descending artery. (Bottom) Electrocardiogram obtained on second presentation demonstrating inferior ST-segment elevations with deep Q waves in leads II, III, and aVF with reciprocal changes in aVL, consistent with a late-presenting inferior myocardial infarction. RCA = right coronary artery; RV = right ventricle.

A 48-year-old man with hypertension, hyperlipidemia, and prior tobacco use presented with recurrent epigastric pain that had been present for 3 days. Similar symptoms had occurred 4 weeks prior, at which time 2 serial non–high-sensitivity troponin I measurements at an outside emergency department were minimally elevated, and his symptoms were attributed to a gastrointestinal etiology. However, the ECG at that visit did show subtle inferior ST-segment changes consistent with an inferior MI, though this was not recognized at the time. His symptoms recurred and progressively worsened over the ensuing weeks. On the second presentation, ECG demonstrated inferior ST-segment elevations with deep Q waves, and high-sensitivity troponin I was 8,276 ng/L and downtrending, consistent with a late-presenting MI (Figure 3).^8^ Coronary angiography revealed a culprit occlusion of the proximal RCA with a large thrombus burden extending throughout the entire vessel (Figure 3). The distal RCA was supplied by collaterals from the LAD. A Runthrough wire was positioned in what was believed to be the distal RCA, then multiple passes of aspiration thrombectomy were performed with an Export catheter (Medtronic) followed by dilation with a 3.0-mm semicompliant balloon. Despite this, there was persistent large thrombus burden with only TIMI flow grade 1 achieved and poor distal outflow (Figure 3). Given the late presentation, downtrending troponin, and Q waves on electrocardiography, the procedure was aborted in favor of intensive antithrombotic therapy with glycoprotein IIb/IIIa inhibition, dual antiplatelet therapy, and unfractionated heparin, with planned repeat angiography in 48 hours.

On repeat angiography, it was evident that the vessel initially treated was a large right ventricular marginal branch; the true RCA remained occluded with extensive thrombus extending into the distal vessel. A blocking balloon technique was employed with a 3.0-mm balloon over a Samurai wire (Boston Scientific) in the right ventricular marginal branch. Despite multiple attempts with Sion, Fielder XT, and Mongo (Asahi) wires, the RCA could not be wired.

**Figure 4:**
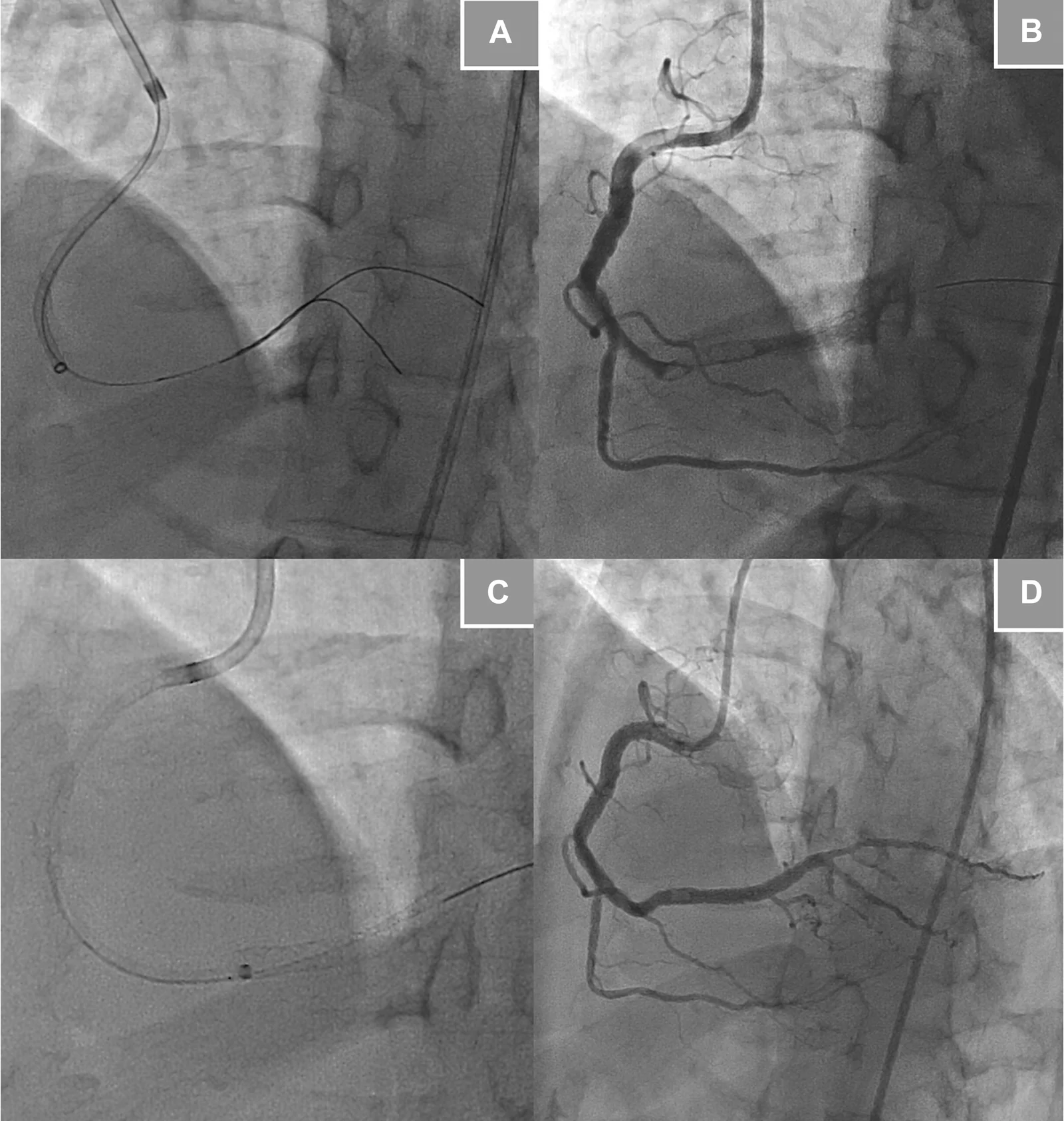
Case 2 Second Coronary Intervention (A) The CAT-7 aspiration thrombectomy catheter advanced into the distal right coronary artery after exchange from the CAT-Rx system, providing more effective aspiration of the extensively organized thrombus burden. (B) Angiography after aspiration thrombectomy and deployment of a 4.0 × 30 mm drug-eluting stent in the distal RCA demonstrating acute stent thrombosis with poor antegrade flow. (C) A 7-F GuideLiner guide extender deep-seated within the stent connected to the CAT-Rx aspiration system performing aspiration thrombectomy for acute stent thrombosis. (D) Final angiography after aspiration thrombectomy and deployment of a 4.5 × 26 mm drug-eluting stent in the proximal RCA demonstrating TIMI flow grade 3 throughout the RCA. RCA = right coronary artery.

HDR was then performed using a Mongo wire and a Turnpike LP.^1^ The Mongo wire was advanced through the contrast-modified channel and crossed into the distal RCA on the first attempt. Intraluminal wire position was confirmed by distal tip injection. Aspiration thrombectomy was then performed with a 6-F CAT-Rx catheter (Penumbra) followed by angioplasty with a 3.0 × 20 mm noncompliant balloon. Given significant residual thrombus in the large-sized distal RCA, the aspiration system was exchanged for a CAT-7 aspiration thrombectomy catheter, which was significantly more effective (Figure 4).^9^^,^^10^ A 4.0 × 30 mm Onyx Frontier drug-eluting stent (Medtronic) was deployed in the distal RCA; however, subsequent angiography demonstrated acute stent thrombosis (Figure 4).^11^ Successful aspiration thrombectomy was then performed through a GuideLiner guide extender (Teleflex) deep-seated within the stent in a 7-F catheter connected to the CAT-Rx aspiration system (Figure 4). The proximal RCA was subsequently stented with a 4.5 × 26 mm Onyx Frontier drug-eluting stent. After intracoronary vasodilator administration, final angiography demonstrated TIMI flow grade 3 in the RCA, posterolateral branch, and large right ventricular marginal branch.

The patient tolerated the procedure without complication. Positron emission tomography performed 3 months later demonstrated only a small inferior nontransmural scar involving 5% of the left ventricle in the distribution of the atrioventricular branch of the RCA, and the patient had returned to his baseline functional status.

### Case 3: Chronic total occlusion in multivessel disease precipitating cardiac arrest from demand ischemia

**Figure 5:**
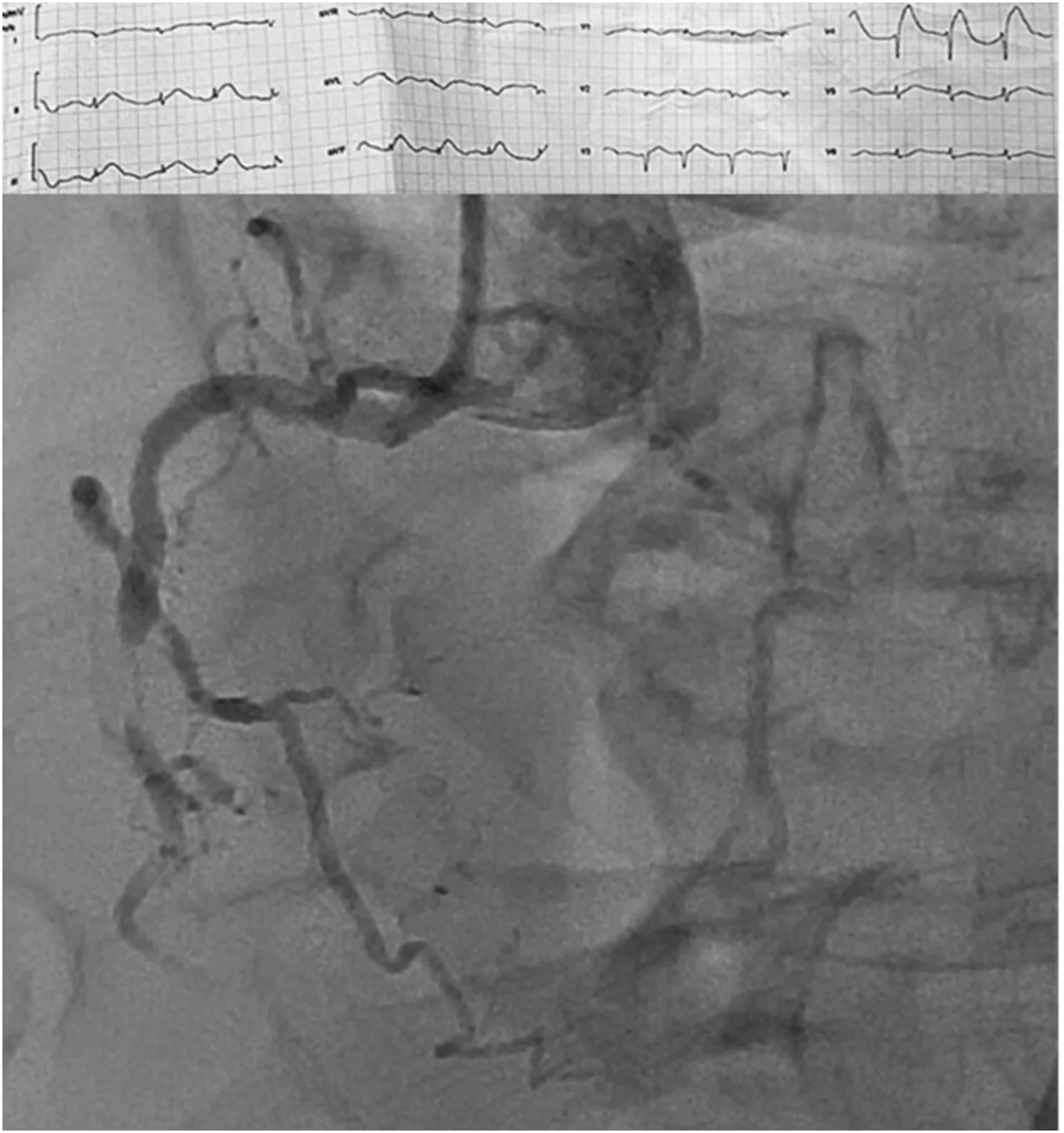
Case 3 Presenting Electrocardiogram and Diagnostic Coronary Angiography (Top) Electrocardiogram obtained after return of spontaneous circulation demonstrating inferior ST-segment elevations consistent with an inferior ST-segment elevation myocardial infarction. (Bottom) Coronary angiography demonstrating chronic total occlusion of the mid-RCA. The distal RCA was primarily filled retrograde by collaterals from the left and right coronaries, reflecting the chronic nature of the occlusion. RCA = right coronary artery.

A 66-year-old man with hypertension and type 2 diabetes mellitus experienced sudden cardiac arrest while performing yard work. Bystander cardiopulmonary resuscitation was initiated, and emergency medical services found the patient in ventricular fibrillation. After defibrillation, brief return of spontaneous circulation was achieved, and an ECG was concerning for inferior STEMI (Figure 5). He subsequently re-arrested and remained in refractory ventricular fibrillation on arrival to the hospital. He was cannulated for femoral-femoral venoarterial extracorporeal membrane oxygenation (VA-ECMO),^12^ and once ECMO flow was established at 4 L/min, the patient was taken emergently to the cardiac catheterization laboratory. Initial high-sensitivity troponin was 102 ng/L, rising to 49,873 ng/L on subsequent measurement.

Coronary angiography demonstrated a total occlusion of the mid-RCA with well-established distal RCA filling via right- and left-sided collaterals (Figure 5). The left coronary system demonstrated moderate to severe diffuse disease with focal stenoses up to 80% but maintained TIMI flow grade 3 without an identifiable culprit lesion (Video 1). The mid-RCA occlusion was identified as the culprit (Figure 5); it was felt that the high-intensity work in the presence of the underlying multivessel coronary artery disease including the RCA CTO likely triggered ischemia-mediated ventricular tachycardia. A Runthrough wire met significant resistance on the first crossing attempt (Video 2). Multiple subsequent wires including a Fielder XT and Sion were attempted; however, even with a deeply seated TrapLiner guide extension catheter (Teleflex) and a Turnpike LP for support, the lesion could not be crossed, consistent with the CTO nature of the lesion.

**Figure 6:**
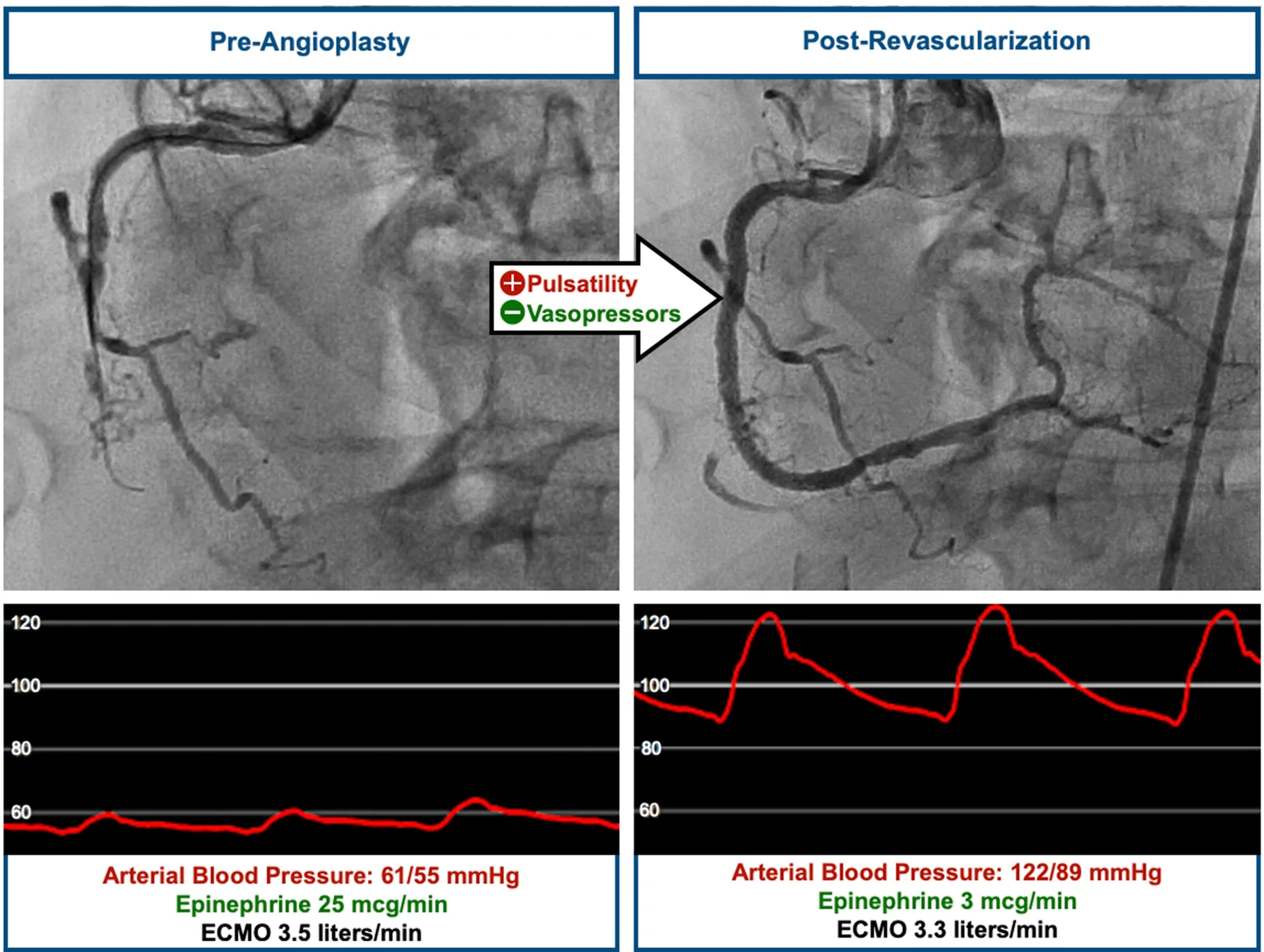
Case 3 Hemodynamic Response to Revascularization Angiography before balloon angioplasty (left) and shortly after restoration of antegrade flow with balloon angioplasty of the mid-to-proximal right coronary artery (right). The angioplasty resulted in near-immediate return of cardiac pulsatility and a dramatic reduction in vasopressor requirements.

HDR was performed using a Fielder XT wire through the Turnpike LP in the proximal cap of the plaque. After limited, low-pressure contrast injection as previously described, the Fielder XT successfully crossed. Balloon angioplasty was performed with a 3.0 × 15 mm noncompliant balloon. Immediately after angioplasty, the patient's hemodynamics improved, with incremental increase in systemic pulsatility (Figure 6) in the presence of fixed ECMO and inotropic support. IVUS was performed and demonstrated a predominantly fibrocalcific occlusion with regions of fatty plaque and circumferential calcification. A 3.0 × 32 mm and a 2.75 × 32 mm Synergy drug-eluting stent were deployed in the mid to proximal RCA and postdilated with a 3.0 × 15 mm noncompliant balloon. IVUS confirmed adequate stent expansion, and final angiography demonstrated TIMI flow grade 3 throughout the RCA.

After successful revascularization, hemodynamics normalized, and inotropes/vasopressors were rapidly weaned within 24 hours. The patient was successfully weaned from VA-ECMO on hospital day 3. Neurological status was intact throughout the hospitalization, and no recurrent arrhythmias were observed. Staged revascularization of the LAD was performed after decannulation. At the 1-month follow-up, the patient remained asymptomatic, left ventricular ejection fraction was 45% to 50%, and the patient had returned to baseline activity level.

## Discussion

HDR leverages the differential interaction of contrast with the heterogeneous components of a coronary occlusion to prepare the path of least resistance through the occlusion for wire crossing. Each of the 3 cases highlights distinct mechanisms and advantages of HDR across the full spectrum of occlusion types.

**Figure 7:**
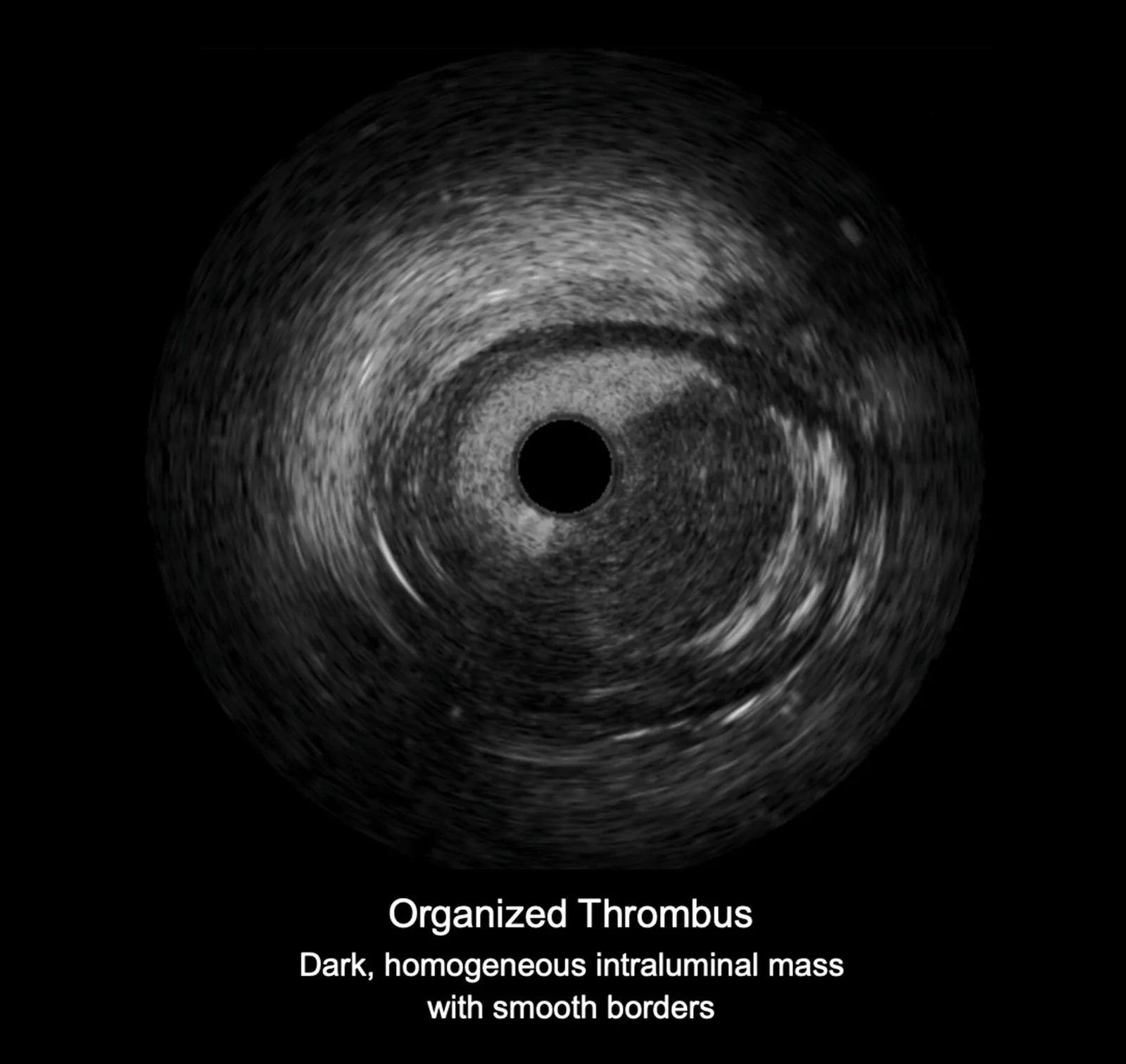
Intravascular Ultrasound Demonstrating Thrombus Composition in Case 1 Intravascular ultrasound imaging of the proximal left circumflex artery following wire crossing and balloon angioplasty. (Left) Acute thrombus appears as a bright intraluminal mass with well-delineated borders and no acoustic shadowing (red arrows), consistent with a fresh, fibrin-poor platelet-rich thrombus. (Right) Early organizing thrombus (asterisk) appears as a gray intraluminal mass with a speckled echotexture and irregular borders (outlined in white), reflecting early fibrin crosslinking.

In case 1, the acute thrombotic occlusion had begun organizing because of the delay for stroke work-up (Figure 7).^13^ The delay made timely reperfusion even more critical, but multiple guidewires with different coatings and tip designs were unable to create or track through a channel in the occlusion using conventional wiring techniques. In HDR, contrast preferentially interacts with the softer, less fibrin-rich portions of thrombus in acute occlusions, which creates a low-resistance, contrast-saturated pathway for the polymer-jacketed wire to track through. Given the acuity of this occlusion, the controlled hydraulic force of the gentle contrast injection disrupted friable thrombus, further preparing the path for wire crossing.

**Figure 8:**
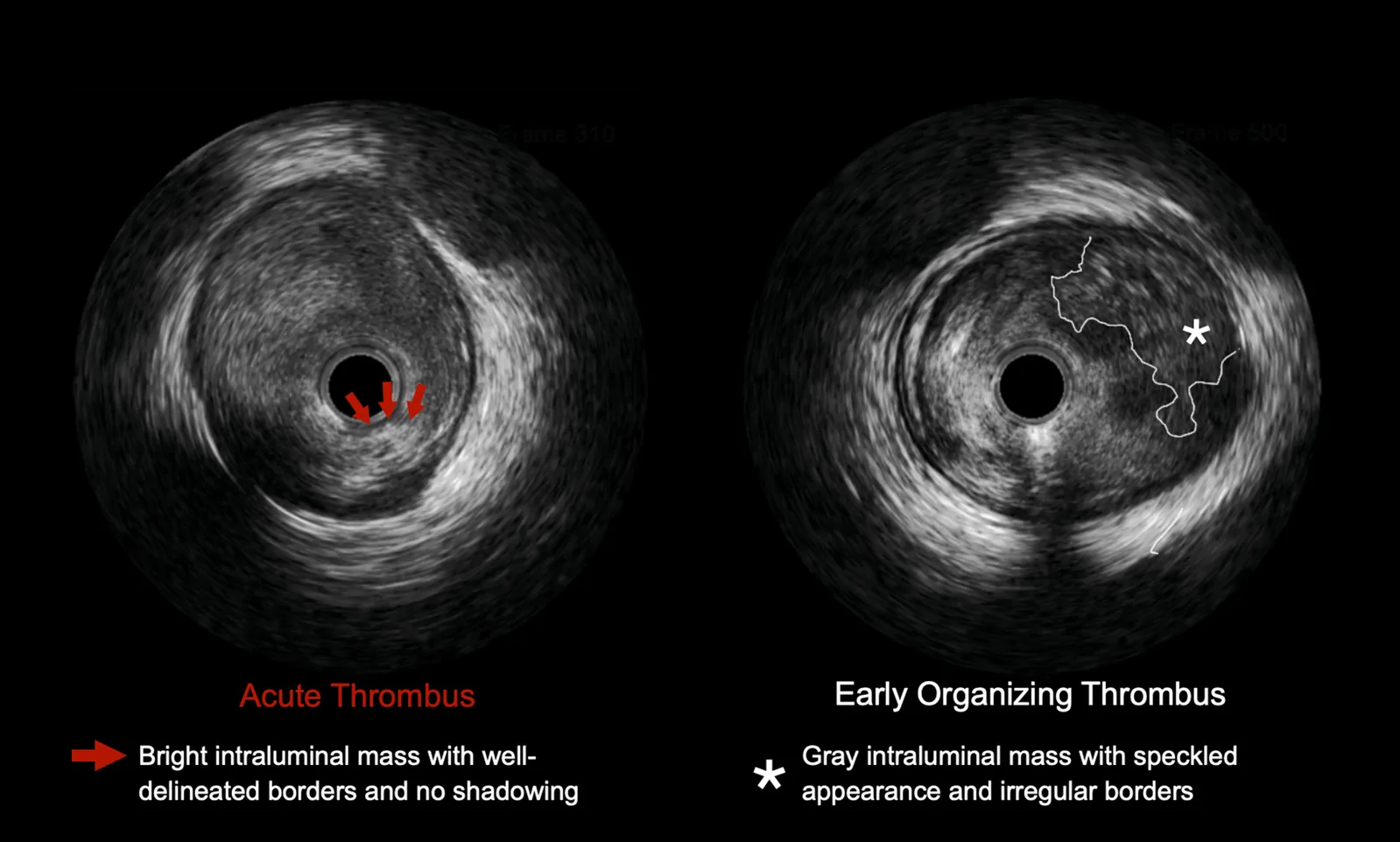
Intravascular Ultrasound Demonstrating Well-Organized Thrombus in Case 2 Intravascular ultrasound imaging of the right coronary artery demonstrating near-circumferential organized thrombus appearing as a dark, homogeneous intraluminal mass with smooth borders characteristic of densely organized, fibrin-rich thrombus.

In case 2, because of the late presentation of the MI, there was extensive organization and propagation of the thrombus from the proximal plaque rupture throughout the entire vessel. This created very poor distal visualization, compounded by unknown vessel anatomy, which initially led to the inadvertent angioplasty of the right ventricular marginal branch. Thrombus can be heterogeneous with varying degrees of organization, especially in cases with such a large thrombus burden. In subacute occlusions, there are often areas of dense organized thrombus (Figure 8),^13^ which can be very challenging to cross, interspersed among areas of softer thrombus; however, it is difficult to differentiate between these angiographically. Blindly probing with a wire to navigate the long occlusion risks vessel injury. With HDR, contrast can safely find and prepare a pathway through the occluded vessel even when there is poor distal visualization. Furthermore, by taking the low-resistance path through such occlusions, HDR may avoid dislodging and embolizing organized portions of thrombus.

In case 3, the “culprit” occlusion was a classic CTO with a hard proximal cap, resisting multiple wires even with a deeply seated guide extension catheter and microcatheter support. Despite the chronic nature of the occlusion, the inferior STEMI presentation and dramatic hemodynamic improvement after revascularization identified this lesion as responsible for the majority of the ischemia given the insufficient degree of left-to-right collaterals in the setting of severe multivessel disease and the increased oxygen demand during exertion. CTO revascularization is rarely required for management of STEMIs, but the immediate return of pulsatility on ECMO with revascularization illustrates the potentially lifesaving improvement in ischemia possible with carefully selected CTOs.^14^

## Conclusions

HDR is a readily adoptable technique that can be safely implemented by non-CTO operators to cross resistant occlusions when conventional wiring strategies fail. Although there may be concern that using HDR in acute MI may prolong time to revascularization, early use when crossing proves difficult can save time, avoid wiring-induced dissection, and may reduce thrombus embolization. This series demonstrates that HDR is applicable across the full spectrum of occlusion acuity.

**Equipment List:** Equipment Used Across Cases

Category	Case 1	Case 2	Case 3
Guide catheter/support			
Guide catheter	EBY 3.75	7 F JR4	6 F JR4
Microcatheter	Turnpike LP (Teleflex)	Turnpike LP (Teleflex)	Turnpike LP (Teleflex)
Guide extension catheter	—	GuideLiner (Teleflex)	TrapLiner (Teleflex)
Wires attempted			
Wires	Runthrough (Terumo) Sion (Asahi) Sion Black (Asahi)	Runthrough (Terumo) Samurai (Boston Scientific) [support wire] Sion (Asahi) Fielder XT (Asahi) Mongo (Asahi)	Runthrough (Terumo) Fielder XT (Asahi) Sion (Asahi)
HDR equipment			
HDR wire	Fielder XT (Asahi)	Mongo (Asahi)	Fielder XT (Asahi)
Microcatheter	Turnpike LP (Teleflex)	Turnpike LP (Teleflex)	Turnpike LP (Teleflex)
Contrast agent	Visipaque (iodixanol)	Visipaque (iodixanol)	Visipaque (iodixanol)
PCI			
Cutting balloon	3.5 × 10 mm Wolverine (Boston Scientific)	—	—
Aspiration catheter	—	Penumbra CAT-Rx (Penumbra) Penumbra CAT-7 (Penumbra) GuideLiner 7-F + CAT-Rx (Teleflex)	—
Stents deployed	4.0 × 20 mm Synergy DES (Boston Scientific) proximal LCx	4.0 × 30 mm Onyx Frontier DES (Medtronic) Distal RCA 4.5 × 26 mm Onyx Frontier DES (Medtronic) Proximal RCA	3.0 × 32 mm Synergy DES (Boston Scientific) 2.75 × 32 mm Synergy DES (Boston Scientific) Midproximal RCA
IVUS catheter	OPTICROSS HD (Boston Scientific)	OPTICROSS HD (Boston Scientific)	OPTICROSS HD (Boston Scientific)

DES = drug-eluting stent; HDR = hydrodynamic contrast recanalization; IVUS = intravascular ultrasound; LCx = left circumflex artery; PCI = percutaneous coronary intervention; RCA = right coronary artery.

## Funding Support and Author Disclosures

Dr Arain has received consulting/speaker honoraria from Boston Scientific, Canon USA, and Teleflex, and he serves on the advisory board of Boston Scientific. All other authors have reported that they have no relationships relevant to the contents of this paper to disclose.

**Take Home Messages**

• To recognize that culprit occlusions in acute MI can be heterogeneous, composed of thrombus with varying degrees of organization among fatty, fibrous, and calcific plaque components, and may resist conventional wiring strategies.
• To understand the mechanism and technique of hydrodynamic contrast recanalization (HDR), in which contrast highlights and prepares the path of least resistance through an occlusion for wire crossing.
• To appreciate that HDR, a technique traditionally used for CTOs, can facilitate wire crossing in acute MI when initial attempts fail, potentially reducing ischemic time, vessel injury, and thrombus embolization.

## Appendix

Video 1 Coronary Angiography in Anteroposterior Projection Left coronary angiography in anteroposterior projection demonstrating moderate to severe diffuse disease of the left coronary system with focal stenoses up to 80% and preserved TIMI flow grade 3 without an identifiable culprit lesion. Retrograde collateral filling of the distal right coronary artery by left-sided collaterals is seen.

Video 2 Coronary Angiography in Right Anterior Oblique Projection Right anterior oblique projection demonstrating a Runthrough guidewire unable to advance through the proximal cap of the mid-RCA chronic total occlusion, consistent with a hard fibrous cap resistant to conventional wire crossing techniques, prompting the decision to employ hydrodynamic contrast recanalization.
